# Monoclonal antibody as a targeting mediator for nanoparticle targeted delivery system for lung cancer

**DOI:** 10.1080/10717544.2022.2120566

**Published:** 2022-09-09

**Authors:** Nasrul Wathoni, Lisa Efriani Puluhulawa, I Made Joni, Muchtaridi Muchtaridi, Ahmed Fouad Abdelwahab Mohammed, Khaled M. Elamin, Tiana Milanda, Dolih Gozali

**Affiliations:** a Department of Pharmaceutics and Pharmaceutical Technology, Faculty of Pharmacy, Universitas Padjadjaran, Sumedang, Indonesia; b Functional Nano Powder University Center of Excellence (FiNder U CoE), Universitas Padjadjaran, Sumedang, Indonesia; c Department of Physics, Faculty of Mathematics and Natural Sciences, Universitas Padjadjaran, Sumedang, Indonesia; d Department of Pharmaceutical Analysis and Medicinal Chemistry, Faculty of Pharmacy, Universitas Padjadjaran, Sumedang, Indonesia; e Department of Pharmaceutics, Faculty of Pharmacy, Minia University, Minia, Egypt; f Graduate school of Pharmaceutical sciences, Kumamoto University, Kumamoto, Japan; g Global Center for Natural Resources Sciences, Faculty of Life Sciences, Kumamoto University, Kumamoto, Japan; h Departement of Pharmaceutical Biology, Faculty of Pharmacy, Universitas Padjadjaran, Sumedang, Indonesia

**Keywords:** Active targeting, drug delivery, lung cancer, monoclonal antibodies, nanoparticles

## Abstract

Lung cancer is the second most common type of cancer after breast cancer. It ranks first in terms of mortality rate among all types of cancer. Lung cancer therapies are still being developed, one of which makes use of nanoparticle technology. However, conjugation with specific ligands capable of delivering drugs more precisely to cancer sites is still required to enhance nanoparticle targeting performance. Monoclonal antibodies are one type of mediator that can actively target nanoparticles. Due to the large number of antigens on the surface of cancer cells, monoclonal antibodies are widely used to deliver nanoparticles and improve drug targeting to cancer cells. Unfortunately, these antibodies have some drawbacks, such as rapid elimination, which results in a short half-life and ineffective dose. As a result, many of them are formulated in nanoparticles to minimize their major drawbacks and enhance drug targeting. This review summarizes and discusses articles on developing and applying various types of monoclonal antibody ligand nanoparticles as lung cancer target drugs. This review will serve as a guide for the choice of nanoparticle systems containing monoclonal antibody ligands for drug delivery in lung cancer therapy.

## 1. Introduction

The lungs are an important organ in the human body, particularly in the respiratory system. Damage to this organ can endanger lives and perhaps result in death. Lung cancer is a form of cancer that affects the human lungs (Bade & Dela Cruz, 2020). This malignancy is the second most common after breast cancer and has the greatest fatality rate of any type of cancer (International Agency for Research on Cancer (IARC), 2020). It is reported that this cancer has a mortality rate of 1,796,144 or 18% of the total number of cancer deaths and an incidence rate of 2,206,771 which is 11.4% of all cancer incidences worldwide both in women and men (Globocan, 2020). There are currently three options for cancer treatment: surgery, radiation therapy, and chemotherapy (Abbas & Rehman, 2018). Stage I or II Non-Small Cell Lung Cancer ‘NSCLC’ treatment is surgical resection of the tumor followed by adjuvant therapy. When the cancer progresses to stage III or IV, the treatments are chemotherapeutic and/or radiation therapy. Since the cancer invaded surrounding tissues, metastases can occur through the circulatory system or lymphatic system (Huang et al., 2015). Chemotherapy is a form of cancer treatment that employs medications. As a result of the drug’s inability to target specific cells, this therapy is often associated with severe adverse effects (Ohnoshi et al., 1992; Partridge et al., 2001; Sun et al., 2005; Aslam et al., 2014). It has inspired the development of cancer medicines, one of which is the use of nanoparticles.

The particle size of nanoparticles in drug delivery systems ranges from 1 to 1000 nanometers (1–1000 nm) (Jeevanandam et al., 2018; Naito et al., 2018; Khan et al., 2019). Nanoparticles are often utilized in the healthcare domain for diagnostic and therapeutic applications (Jiang et al., 2008; Sukhanova et al., 2018). This approach has a number of benefits in cancer therapy, including improving drug bioavailability through increased dissolution rate (Li et al., 2015; Jafari & McClements, 2017), minimizing pharmacological adverse effects via modest dosages, and maintaining constant drug levels in plasma (Park et al., 2009). As a diagnostic agent, nanoparticles possess qualities such as size, optical properties, photodynamic magnetic properties, and others that can aid in the diagnosis of cancer (Al-Jamal & Kostarelos, 2007; Fang & Zhang, 2010; Wu et al., 2019).

Nanoparticles as a passive delivery route for cancer therapy, particularly lung cancer, have been widely studied (Ye et al., 2008; Bazak et al., 2014; Clemons et al., 2018). Alternative nanoparticle delivery strategies for lung cancer have also been developed. Based on the interaction of certain ligands and receptors that are abundantly expressed in cancer cells, this targeting mechanism is able to specifically identify cancer cells (You et al., 2008; Bazak et al., 2015; Costa et al., 2019). Up to this point, there have been no therapies that transport drug directly to the tumor site (Stella et al., 2000; Steinhauser et al., 2006; Xu et al., 2019). In this active nanoparticle delivery technique, monoclonal antibodies are one of the ligands used for this purpose.

Monoclonal antibodies are currently being used extensively to deliver nanoparticles to multiple antigens on the surface of cancer cells. This antigen is what distinguishes cancerous cells from healthy ones, because their expression is higher in cancer cells, drug-targeting therapies can take advantage of this. Due to enhanced drug targeting, the systemic toxicity of the drug treatment is reduced by conjugating monoclonal antibodies to the nanoparticles (Trail & Bianchi, 1999; Adams & Weiner, 2005). Generally, different receptor expressions were found in cancer cells. Monoclonal antibodies are aimed solely to target receptors such as the Epidermal Growth Factor Receptor, EpCAM receptor, NSE receptor, and other receptors that are abundantly expressed in lung cancer (Tseng et al., 2007; Wang & Zhou, 2015; Chen et al., 2021). Unfortunately, it has a major drawback, including fast clearance from circulation, which results in a short half-life and renders the dosage ineffective. As a result, monoclonal antibodies are still synthesized in nanoparticles to improve their targeting ability and reduce their disadvantages (Silvestre et al., 2020).

Monoclonal antibodies have been conjugated in several nanoparticle systems, including lipid-based nanoparticles, gold nanoparticles, super magnetic iron oxide nanoparticles, and other materials for active lung cancer targeting. The objective of this linkage is to explore remedies for this type of cancer. Therefore, this review summarizes and discusses the deployment of several kinds of nanoparticles in combination with ligand monoclonal antibodies to target lung cancer cells.

## 2. Methodology

**Figure 1.**
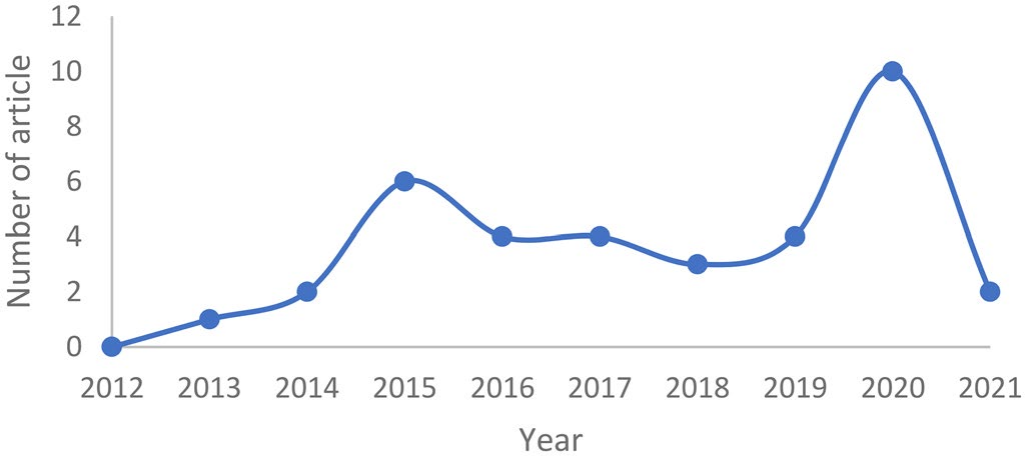
Number of articles used by year.

**Figure 2.**
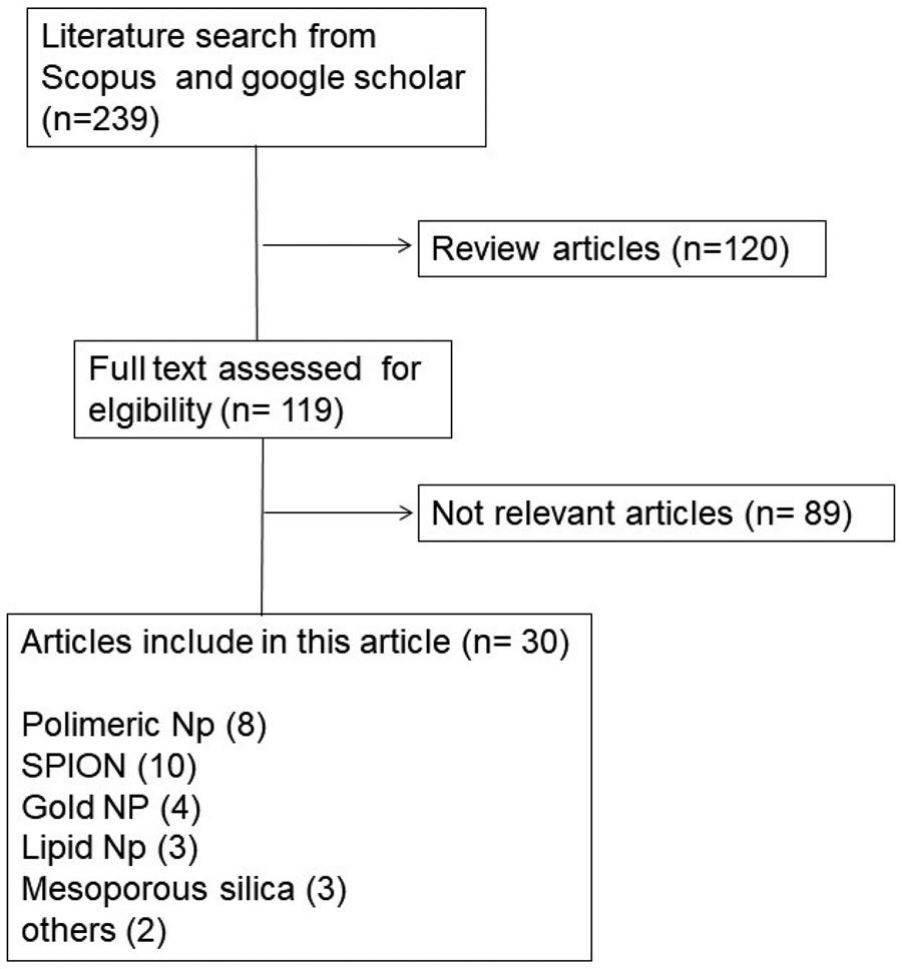
Flowchart of methodology.

The publications were identified using the keywords ‘monoclonal antibody nanoparticle for lung cancer’, ‘anti-EpCAM nanoparticle for lung cancer’, ‘anti-EGFR nanoparticle for lung cancer’, in Scopus and Google Scholar databases. The selection of these journals was subjected to inclusion and exclusion criteria. Articles published in the recent 10 years (2012–2021) were included in the study, while review articles were excluded. There were 239 articles in our initial search. Some, such as the formation of nanoparticles with monoclonal antibody ligands for lung cancer treatment, were removed since they might not meet the criteria. As a result, the total number of articles utilized in this evaluation is 36, with the most articles being published in 2020. (Figure 1). The process is depicted in (Figure 2) as a flowchart.

## 3. Targeted drug delivery

### 3.1. Passive targeting drug delivery strategy

As a strategy, passive targeting relies on the tumor microenvironment for improved permeability and retention effects. Through the retention effects of nanoparticles in the circulation, it is possible to be localized within cancer tissue, thus facilitating the accumulation of drugs into cancer tissue, avoiding systemic metabolism, which is widely utilized in cancer therapy (Won et al., 2012; Wakaskar, 2017).

The passive targeting of nanocarriers is based on their biodistribution in the body, where these nanoparticles can be rapidly cleared from the body due to being rapidly opsonized and engulfed by macrophages. However, when the rapid cleaning of the nanoparticles is minimized, there is a significant increase in their bioavailability. Accumulation of nanoparticles in solid tumors through the phenomenon called increased permeability and retention effect (Szczepanowicz et al., 2016).

Enhanced permeability and retention (EPR) occurs because a limited amount of fluid is supplied to the lymphatic circulation, blood capillaries in injured tissues become more permeable [39–40]. Nanoparticles can deliver the medicine to the tumor site in a concentrated form since they are tiny and small enough to travel (400 nm or smaller). A variety of angiogenesis-regulating substances, such as vascular endothelial growth factor (VEGF), which are widely expressed in tumors, have the capacity to physiologically alter tumor vessel shape as well as increase vascular permeability (Kreuter, 2007; Alavi & Hamidi, 2019).

### 3.2. Active targeting drug delivery strategy

The drug delivery system utilizes two distinct mechanisms: passive targeting based on increased permeability and retention, and active targeting based on the ligand binding to its receptor (Salahpour Anarjan, 2019). The active targeting system is one of the drug targeting strategies using the help of a ligand in which this ligand will bind to its specific receptor (Byrne et al., 2008). The ligand will be conjugated on the surface of the nanoparticles resulting in increased cellular uptake by receptor-mediated endocytosis, hence increased drug accumulation in cancer cells (Li et al. 2013). This mechanism relies on the interaction between conjugated ligands on the surface of nanoparticles and cell surface receptors or antigens on the surface of cancer cells (Muhamad et al., 2018; Yao et al., 2020). The inclusion of ligands to this targeting mechanism allows nanoparticles to be delivered to precisely identified cells or even subcellular locations, and therefore decreasing cytotoxic drug systemic exposure (Maeda & Matsumura, 1989; Yu et al., 2010).

There are several biological ligands that have been found which are known to improve active nanoparticle targeting systems. When a ligand binds to a receptor on the cell surface, the amount of drug accumulated increases, as does the effectiveness of the treatment. Ligands have been identified to be proteins, polysaccharides, nucleic acids, peptides, and small compounds. Nanoparticles and ligands will interact in two distinct ways, namely at the stage after nanoparticle fabrication, chemical conjugation, or physical entrapment of nanoparticles, and the second at a stage prior to nanoparticle production, through binding to nanoparticle components such as polymers (Yoo et al., 2019).

The most important challenge in active targeting systems is determining the best agent(s) to be used to deliver the nanoparticle system to cancer tissue while avoiding toxicity. Furthermore, the targeted agent or ligand must have high affinity for the cancer cell surface to trigger endocytosis. This interaction will transport the therapeutic agent to the cancerous site (Wakaskar, 2017).

**Figure 3.**
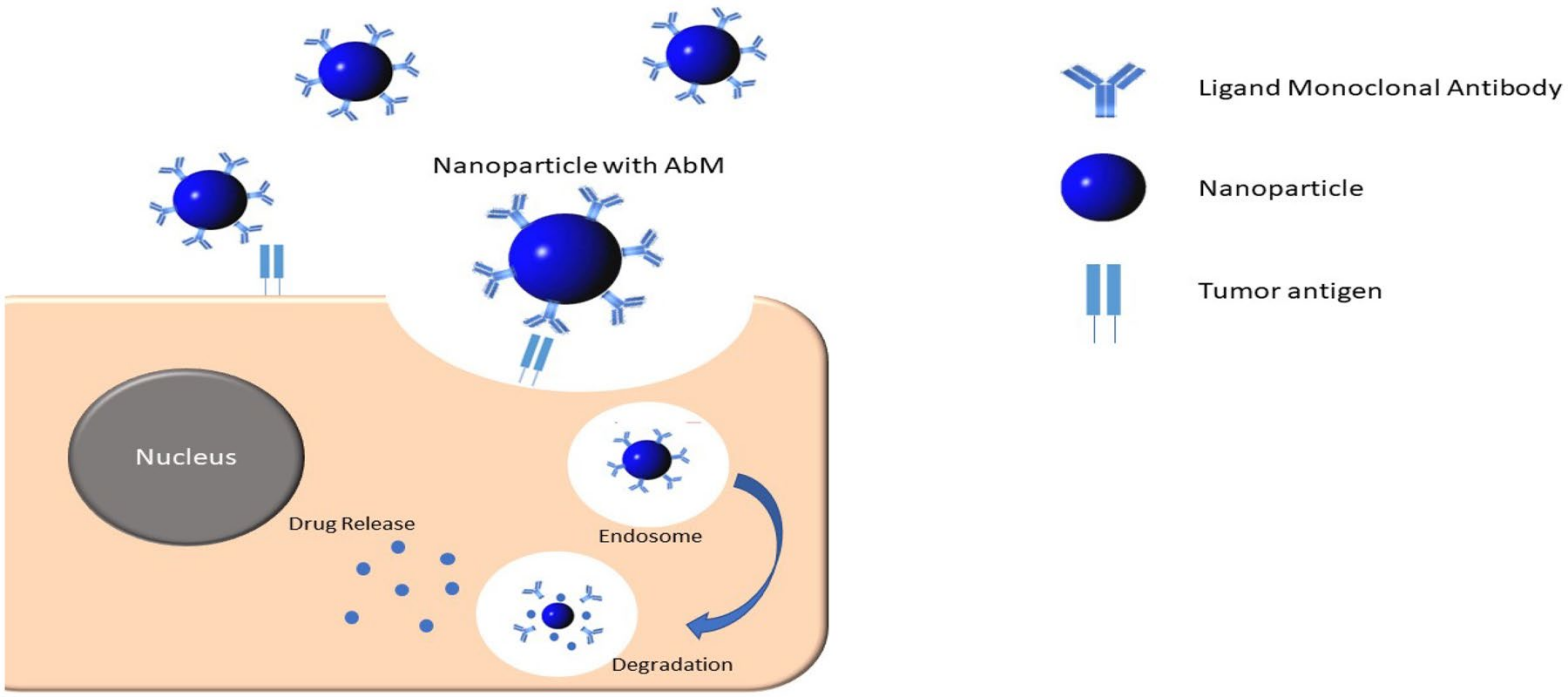
Targeted drug delivery system using monoclonal antibody.

This targeted delivery technique is categorized into three parts: organ-targeted, cell-targeted, and subcellular-targeted. The organ-targeted delivery method is designed to distribute the drug into an organ by taking use of the organ’s unique characteristics. For example, the liver possesses tissue properties that allow macromolecules and microparticles that easily infiltrate it, ensuring that the drug does not damage adjacent tissues due to massive connections. The cell-targeted delivery system includes a recognizable substance that binds to corresponding antigens and receptors on the cell surface. The subcellular delivery system delivers drugs to specific locations within the cells, such as the nuclei of cells (Figure 3) (Winarti, 2013).

## 4. Receptor of monoclonal antibody on lung cancer

Cancer cells are cells with a high rate of proliferation. This property causes the expression of several receptors that activate proliferative activity in these cells much more than in normal cells. Researchers have exploited this receptor overexpression in developing active targeted drug delivery systems.

Several receptors, including the Epidermal Growth Factor (EGFR) receptor, are overexpressed in lung cancer, including HER-1, HER-2/neu, HER-3, and HER-4. Furthermore, cell membrane receptors with intrinsic tyrosine kinase activity can change proliferative signals in response to various binding ligands. EGFR overexpression is associated with increased tumor proliferation, poor differentiation, a high probability of metastasis, and a poor prognosis in these cancers (Seyhan et al., 2010). The expression of this receptor in ‘NSCLC’ is estimated at 32% (Rusch et al., 1997).

Another widely expressed receptor on lung cancer cells is the Epithelial Cell Adhesion Molecules (EpCAM) receptor, which is a receptor responsible for mediating the adhesion of epithelial-specific, Ca2+-independent homotypic cells and represents the first tumor-associated antigen discovered in humans (Pak et al., 2012). The expression of these receptors is associated with an increase in tumor progression. Recently, the EpCAM-specific therapeutic drug was licensed for clinical usage in cancer patients (Spizzo et al., 2011). Expression of this receptor in NSCLC was reported as 51.3% in adenocarcinoma tissue (Kim et al., 2009).

The NSE receptor or Neuron Specific Enolase is another receptor that is also overexpressed in lung cancer (Tiseo et al., 2008). Previously, this receptor was considered as a biomarker for small cell lung cancer ‘SCL’. The expression of this NSE receptor is associated with the process of cancer metastasis, with the outcomes of functional study revealing that overexpression of this NSE receptor increases the migration and invasion of SCL cells (Zha et al., 2021). Other receptors are also found in lung cancer such as disialoganglioside receptors of GD2, GD3, and so on (Tivnan et al., 2012).

## 5. Nanoparticle with monoclonal antibody for lung cancer

Polymeric nanoparticle systems, lipid-based nanoparticles, dendrimers, etc have been utilized to prepare nanoparticles containing ligand monoclonal antibodies for lung cancer therapy. The utilization of these various types of nanoparticles for lung cancer is presented in Table 1.

**Table 1.** Applying Nanoparticles with Ligand Monoclonal Antibodies for Lung Cancer.

No.	Types of Nanoparticles	NDDS	Ligands	*In-vitro* and *In-vivo* Model	Activities		Ref
					CT	CU	
1.	Polymeric nanoparticle	Doxorubicin, PLGA-b-PEG, RNA	Anti-EpCAM	SK-MES-1 and A549 cell lines; rats	+	+	(Deng et al., 2014)
2.		Demethoxycurcumin, chitosan	Anti-EGFR	A549 cell line; rats	+	+	(Huang et al., 2015)
3.		Docetaxel, poly(lactide-co-glycolide)	Anti-EGFR	A549 cell line; rats	+	+	(Zhang et al., 2018)
4.		Doxorubicin, hyaluronic acid, and amphipathic cationic starch	Anti-EGFR (erlotinib apatinib and icotinib	A549, NCI-H1975, and PC9 cell lines; rats with NSCLC	+	+	(Malhotra et al., 2021)
5.		Doxorubicin, PLGA, LFC131	Anti-CXCR4	A549 cell line	+	+	(Lin et al., 2019)
6.		Paclitaxel palmitate, PLGA, oleyl cysteine amide	Anti-EGFR	A549 cell line; rats induced by A549 cell	+	+	(Li et al., 2018)
7.		Gemcitabine, PLA	Anti-EGFR	A549 cell line	+	+	(Wang & Zhou, 2015)
8.		Docetaxel, Chitosan	Anti-Tn	A549 cell line	+	+	(Alibolandi et al., 2015)
9.	Gold nanoparticle	AuNP	Anti-EGFR (cetuximab)	A431 cell line; C57BL/6 mice; dan pada nude mice with A431 cancer cells	+	+	(Patel et al., 2018)
10.		AuNP	Anti-EGFR	A549 cell line; rats	+	+	(Li et al., 2020)
11.		Gold-decorated polyaniline derivatives (Au-PANI derivatives) include poly-gold(o-aminophenol) (Au-PoAP) and poly-gold (p-phenylenediamine) (Au-PpPD)	Anti-CEA, anti-CYFRA21-1 dan neuron-specific enolase (NSE),	Lung Cancer	+	–	(Karra et al., 2013)
12.		Tetrachloroauric acid, docetaxel, PEG, AuNP	Anti-EGFR	In vitro	+	–	(Chittasupho et al., 2014)
13.		(AlPcS4Cl) and AuNPs	Ig Abs, CD133 Ab, CD56 Monoclonal Anti-N Cam and CD44 Ab	A549 cell	+	+	(Castro et al., 2021)
14.		Tetraethyl orthosilicate, aminopropyl triethoxysilane, macrocyclic chelator DOTAGA anhydride, GD3+	Anti-MUC1-C A	H460 cells lines and Balb/c mice	+	+	(Wang et al., 2019)
		Fe 3 O 4 /Au	Anti-EGFR	SPC-A1 cell line and Balb/c mice	+	+	(Corsi et al., 2020)
15.	SPION	Microfluidic silicon nanowire, SPION	Anti-EPCAM	Blood samples of patients with lung cancer	+	+	(Ashton et al., 2018)
16.		SPION	Anti-EGFR	Healthy C57BL/6 experimental animals and cell line LLC1-induced rats	+	+	(Kao et al., 2014)
17.		SPION	Anti-EGFR	LLC1 cell line; experimental animals	+	+	(Wang, Liu, et al., 2015)
18.		PEG, SPION	Anti-EGFR	H460 cell line	+	+	(Crous & Abrahamse, 2020)
19.		PEG, SPION	Anti-EGFR	H460 cell line	+	+	(Detappe et al., 2020)
20.		Albumin, SPION, ShRNA	Anti-EGFR	clg-82 cell line	+	+	(Lu et al., 2021)
21.		Albumin, SPION, plasmid pDONR223-IFNG	Anti-EGFR	GLC-82 cell line	+	+	(Kowalik et al., 2020)
22.		Doxorubicin, SPION	Anti-EGFR	A549 cell line	+	+	(Lammers et al., 2015)
23.		PLGA, SPION, PEG-aldehyde	Anti-EGFR	A549 cell line	+	–	(Yan et al., 2018)
24.		SPION, carboxymethyl dextran	Anti-CD44v6	A549 cell line	+	+	(Wang, Ye, et al., 2015)
25.	Lipid-based Nanoparticle	Oxygen, liposomes	Anti-EGFR	A549, H1975, and PC-9 cell lines	+	+	(Abdi & Shahbazi-Gahrouei, 2021)
26.		Chloroquine, shRNA	Anti-EGFR	H1975 and PC-9 cell lines	+	+	(Haddada et al., 2020)
27.		Adriamycin, cholesterol lecithin, PVA	Anti-PD-L1	A549 cell line; rats induced by A549	+	–	(Shahbazi-Gahrouei et al., 2020)
		Hyaluronic acid, lipid-polymer hybrid nanoparticles	Anti-EGFR (Erlotinib ) and anti-VEGF (bevacizumab	A549 and H1975 cells	+	+	(Wang et al., 2017)
28.	Silica and Mesoporous silica	Graphene oxide, silica	Anti-EGFR	A549 cell line	+	+	(Wang et al., 2017)
29.		Silica, NIRF methylene blue	Anti-EGFR	A549 cell line; rats induced by A549	+	+	(Hou et al., 2016)
30.		siPLK1, PEG	Anti-EGFR	A549 cell line; rats induced by A549	+	+	(Zhang et al., 2015)
31.	Others	RNAi, adamantane-PEG, cyclodextrin-grafted branched polyethylenimine	Anti-EGFR	A549 cell line	+	+	(Zhang et al., 2019)
32.		1,4,7-triazacyclononane-triacetic acid	Anti-CD146	A549, NCI-H358, NCI-H522, HCC4006, H23, and NCI-H460 cells lines	+	+	(Salehnia et al., 2019)
33.		Doxorubicin	Bevacizumab avastin monoclonal antibody	A549 cells line	+	–	(Wan et al., 2016)
34.		Gemcitabine, DP-GEM/PEI-PE	Anti-EGFR	A549 cells line and famale BALB/c nude mice	+	+	(Ninomiya et al., 2014)

**Abbreviations:** NDDS: nanoparticle drug delivery system; CU: cellular uptake; CT: Citotoxicity; (+): increase; (-): no data.

### 5.1. Polymeric nanoparticle

Polymeric nanoparticles are nanoparticle systems in which polymers are used to entrap drugs (Jeevanandam et al., 2018). These polymeric nanoparticles have been widely developed for application of passive and active targeted drug delivery systems, particularly for cancer treatment fields (Deng et al., 2014; Li et al., 2018; Zhang et al., 2018; Lin et al., 2019; Malhotra et al., 2021). There have also been several developments in polymeric nanoparticles for active targeted delivery systems for the treatment of lung cancer (Table 1), one of which makes use of ligands monoclonal antibodies. The extracellular domain of epithelial cell adhesion molecules was targeted using an EpCAM in the study by Alibolandi et al. (2015) using PLGA-b-PEG nanopolymersomes and an RNA aptamer. The SK-MES-1 and A549 cell lines were used to test these nanoparticles. Results demonstrated an increase in the amount of drug absorbed by cells and a rise in the toxicity that drug produced. *In-vivo* results revealed that tumor development was inhibited and decreased tumor volume, size, and density. It demonstrates that EpCAM antibodies can increase the targeting of nanoparticles to lung cancer cells (Alibolandi et al., 2015).

Demethoxycurcumin was entrapped in chitosan nanoparticles coated with anti-EGFR ligands. These nanoparticles were evaluated *in vitro* on the A549 cell line and *in vivo* on rats. The nanoparticles had a particle size of 200 nm as well as a controlled release system. These nanoparticles were effective at delivering drugs to EGFR receptors, resulting in an eight - fold reduction in tumor mass in experimental animals compared to the control group (Huang et al., 2015). PLGA nanoparticles were also successfully formulated to entrap docetaxel with the cetuximab ligand to actively deliver the drug to the EGFR receptor. These nanoparticles were formulated by solvent evaporation method and resulted in nanoparticles with a particle size of 128.4 nm and zeta potential value of −31 mV. *In vitro* results showed that these nanoparticles were able to release 25% of the drug at a pH of 5.5 after 48 hours. In addition, these nanoparticles were able to decrease the viability of A549 cell line and induce apoptosis. As for the *in vivo* result on mice with tumor volume 150 mm^3^, these nanoparticles showed a significant reduction in tumor growth, and the tumor volume decreases about 81% (Patel et al., 2018).

Doxorubicin is encapsulated in polymeric nanoparticles comprised of amphipathic cationic starch and hyaluronic acid. The inclusion of erlotinib, apatinib, and icotinib, which are anti-EGFR ligands, were conducted to be evaluated on A549, NCI-H1975, and PC9 cells line. Rats were used as experimental animals for *in vivo* investigations. When compared to other ligands, the data showed that icotinib was the most effective. Icotinib nanoparticles had a particle size of 65.7 nm, which increased cytotoxic activity and prevented cancer cell types from migrating. The *in vivo* test findings revealed an increase in the concentration of nanoparticles at the target region and enhanced drug selectivity compared to the normal cells (Li et al., 2020). Anti-EGFR was also used to deliver paclitaxel palmitate nanoparticles. Cetuximab administration in this system increased A549-luc-C8 absorption, internalization, and therapeutic efficacy *in vitro* and i*n vivo* in metastasis lung tumor (Karra et al., 2013).

Wang, Liu, et al. (2015) conducted another study in which they used anti-EGFR to deliver gemcitabine nanoparticles to patients with NSCLC. *In vitro* test results performed on A549 cells showed an increase in cellular uptake, observed by measuring the fluorescence intensity of cells treated with anti-EGFR nanoparticles compared to cells treated without-anti-EGFR nanoparticles (Wang & Zhou, 2015). LFC131 is likewise a monoclonal antibody against the CXCR4 receptor, which is highly expressed in lung cancer. LFC131 can enhance the accumulation of doxorubicin nanoparticles made of PLGA polymer and can efficiently deliver drugs to A549 cells (Chittasupho et al., 2014). In another study, Tn antigen was also used in nanoparticle formulation with chitosan polymer to deliver doxorubicin. The results revealed that cellular absorption increased while cell viability decreased. Tn antigen is widely used as antibodies specific and a lung cancer-specific antigen (Castro et al., 2021).

### 5.2. Gold nanoparticle

Gold nanoparticles (AuNP) are an example of nanocarriers exhibiting favorable size, shape, stability, and biocompatibility. Gold nanoparticles’ adjustable surface and distance-dependent optical properties reveal their enormous potential to be used in a variety of scientific domains (Wang et al., 2019). Due to their surface charge or potential zeta value, gold nanoparticles have been used as drug carriers, supported by their physicochemical properties, stability, and incorporation into cellular processes, as well as their further accumulation. The level of toxicity assigned to AuNPs is highly dependent on the surface charge of the particles, with positively charged gold nanoparticles causing cell death at much lower concentrations, while neutrally charged particles cause cell death at much higher concentrations (Corsi et al., 2020).

Gold nanoparticles have been widely developed for drug delivery in lung cancer. Conjugation of gold nanoparticles with ligand monoclonal antibodies for lung cancer has been widely carried out to obtain a precisely targeted delivery system. Asthon et al. (2018) used two distinct ligands to create gold nanoparticles that target EGFR: the cetuximab ligand; and a single-domain llama-derived anti-EGFR antibody with a lower binding affinity than cetuximab. Cetuximab-containing nanoparticles exhibited the lowest residence time *in vitro,* and all nanoparticles accumulated at the tumor site more than control nanoparticles (without ligand). Cetuximab nanoparticles accumulated at a significantly greater rate than any other (Ashton et al., 2018).

The findings of the *in vitro* experiments revealed that the inclusion of ligand improves the uptake of the A549 cell line by 14.9 times when compared to the formulation without ligand. *In vivo* studies performed to determine the biodistribution in rat with tumor volume about 229 mm^3^, revealed that the nanoparticles with cetuximab ligand actively carried the medicine to the experimental animals generated cancer sites (Kao et al., 2014).

The two new redox-active species of gold-decorated polyaniline derivatives (Au-PANI derivatives), poly-gold(o-aminophenol) (Au-PoAP) and poly-gold(p-phenylenediamine) (Au-PpPD), were synthesized as nanoparticles by a one-pot method utilizing oxidants in the form of chloroauric acid and monomers in the form of o-aminophenol The gold nanoparticles exhibited an immunosensor with a wide linearity range from 0.01 to 100 ng/mL and a detection limit of 6.3 pg/mL for CEA, 8.5 pg/mL for CYFRA21-1, and 7.9 pg/mL for NSE. Furthermore, the findings from this immunosensor were compatible with those from the enzyme-linked immunosorbent test (ELISA), suggesting that nanoparticles containing lung cancer biomarkers may detect the presence of lung cancer (Wang, Liu, et al., 2015).

The use of gold nanoparticles with other monoclonal antibody ligands was carried out by Crous and Abrahamse (2020), who succeeded in formulating a photosensitizer (PS) (AlPcS4Cl), AuNPs and Abs with several antibodies used (Ig Abs, CD133 Ab, CD56 Monoclonal Anti-N Cam and CD44 Ab). The results showed that the nanoparticles were well localized in homeostatic cells, and showed good cytotoxicity and cell death activity in AlPcS4Cl-AuNP-Ab A549 cell line compared to AlPcS4Cl (Crous & Abrahamse, 2020). In addition, the formulation of tetraethyl orthosilicate, aminopropyl triethoxysilane, macrocyclic chelator DOTAGA anhydride, GD3+ in the form of gold nanoparticles with anti-MUC1-C ligand suggested that there was an increase in retention *in vivo* and by administering anti-MUC1-C/NPs with XRT (radiation therapy), it was possible to significantly increased the inhibition of tumor growth and to prolong overall animal survival (Detappe et al., 2020). Another conjugation using Fe_3_O_4_/Au with monoclonal antibody EGFR (scFv) showed that scFv was able to deliver Fe_3_O_4_/Au to NSCLC *in vivo* and increased the localization of these nanoparticles to the target site (Lu et al., 2021).

### 5.3. Spion

Super magnetic iron oxide nanoparticles (SPION) are a type of nanoparticle composed of magnetite (Fe_3_O_4_) crystals with a face-centered cubic lattice and an oxygen-based solid foundation. It has octahedral sites parallel to the external magnetic field in ferrous (Fe^2+^) and iron (Fe_3_^+^) ions. In the tetrahedral area opposing the external magnetic field, Fe_3_^+^ occupies a tetrahedral area. (Kowalik et al., 2020) SPION’s physicochemical features include high sensitivity, low toxicity, and the ability to more readily change the surface. (Lammers et al., 2015) SPION has a hydrodynamic diameter of 5-300 nm and is frequently utilized for diagnostic and therapeutic purposes (Lammers et al., 2015; Yan et al., 2018). SPION can be used to deliver and treat lung cancer by passive or active administration employing ligand monoclonal antibodies (Table 1).

Several studies on active targeted delivery of nanoparticles involving this ligand have been widely used (anti-EPCAM and anti-EGFR). Wang, Ye, et al. (2015) developed a method for simultaneous CTC (Circulating Tumor Cells) trapping and detection by merging a silicon nanowire (SiNW) microfluidic array with multifunctional magnetic conversion nanoparticles. These nanoparticles were coupled with anti-EpCAM, allowing them to precisely detect tumor cells in blood samples from metastasis tumor patients when exposed to an external magnetic field. The results demonstrated that this method enabled for the sensitive identification of a small number of tumor cells, which could then be collected for further investigation and re-culture. These nanoparticles have been used to detect CTCs in clinical blood samples of lung cancer patients by monitoring UCL (up conversion luminescence) signals and clinical outcomes of lung cancer metastases. The findings were consistent (Wang, Ye, et al., 2015).

Abdi and Shahbazi-Gahrouei (2021) report on the performance of superparamagnetic iron oxide (SPION) nanoparticles combined with EGFR receptor antibodies for the detection of lung cancer using Magnetic Resonance Imaging (MRI). The study was conducted on C57BL/6 experimental mice utilizing LLC1cell line. The results showed that employing ligand antibodies increased SPION absorption in cancer cells, as evaluated by an increase in atomic absorption spectrophotometry intensity (AAS). It reveals that tagging SPION with a ligand can identify cancerous cells (Abdi & Shahbazi-Gahrouei, 2021).

SPION has also been produced in nanometric complexes with docetaxel and dicarboxylic acid-terminated polyethylene-glycol (PEG). The complex generated metal-hybrid nanoparticles after sodium borohydride (NaBH_4_) reduction, with the active ingredient protected in a gold core contained in the polymer chain. These nanoparticles were coupled with a human anti-EGFR antibody to target the overexpressed hERG1 channel on human lung cancer cell membranes, which increased anticancer efficacy. The findings revealed that three-dimensional (3 D) spheroids formed on the Air-Liquid Interface, mimicing tissues *in vitro*. It was observed that encapsulating docetaxel in the gold core had a substantial impact on pharmacological efficacy, with a large rise in the therapeutic index when docetaxel was conjugated with Au and explicitly targeted against EGFR (Haddada et al., 2020).

SPION conjugation with an anti-EGFR monoclonal antibody has also been accomplished and published. C57BL/6 mice were used to execute ex *vivo* and *in vivo* cytotoxicity studies on Lewis lung cancer cells (LLC1). The findings indicated that these nanoparticles exhibited spherical shapes of 20 and 80 nm in the nanoparticles and SPION-EGFR, respectively. The results of cell viability after 24 hours of incubation with various nanoprobe concentrations indicated just a 20% decrease in cell viability. The nanoprobe was developed and given by systemic injection into C57BL/6 mice (tumor diameter about 4–6 mm) revealed a great absorption in tumors as well as appropriate imaging signal intensity in both ex vivo and in vivo conditions. The nanoprobe concentration was higher in SPION-EGFR compared to SPION without EGFR and control. It recommends that the nanoprobe be given specifically to the tumor site (Shahbazi-Gahrouei et al., 2020).

SPION conjugated with anti-EGFR was also successfully formulated by Wang, Tang, et al. (2017) who expected these nanoparticles to be conjugated via the epidermal growth factor receptor (EGFR). The results of these nanoparticles showed a greater increased in cell death indicated by a significant decrease in the signal intensity of the H460 cell line on T2WI compared to nanoparticles without ligands. These nanoparticles demonstrated that higher intracellular iron (Fe) of the nanoparticles was observed in the H460 cell line compared to the nanoparticles without the ligand. These results suggest that the anti-EGFR ligands can target the nanoparticles to the overexpressed EGFR in the H460 cell line *in vitro* (Wang, Tang, et al., 2017). The subsequent development of SPION with active targeting enhances imaging sensitivity and energy deposition efficiency when used with a clinical MRgFUS (Magnetic Resonance guided Focused Ultrasound) system. For lung cancer targeted delivery with EGFR overexpression, the surface of these PEGylated SPION nanoparticles has been coated with an anti-EGFR. These nanoparticles were studied *in vitro* and *in vivo* in a human lung cancer xenograft mouse model (H460). This study found that, as compared to SPION without ligand, SPION with ligand had superior targeting ability against H460 tumor cells. Furthermore, SPION in combination with ligands may considerably improve the efficiency of ultrasonic energy deposition in MRgFUS in *in vivo* model (Wang, Qiao, et al., 2017).

The formulation using magnetic albumin immuno-nanospheres (MAINs) loaded simultaneously with SPION to entrap plasmid-survivin/shRNA (pshRNA) in the form of an anticancer gene, and modified by the anti-EGFR cetuximab to improve the targeting mechanism has been studied. The *in vitro* release profile test used in the research demonstrated that nanospheres had an impact on pshRNA release. According to the results of the agglutination test and immunofluorescence analysis, the immunonanosphere sustained Cetuximab’s immunological reactivity. By manipulating magnetic albumin nanospheres, the MAIN significantly increased the adhesion and absorption of GLC-82 lung cancer cells overexpressing EGFR (MANs). The MANs formulation with pshRNA outperformed equimolar doses of Cetuximab, and single magnetic targeting with pshRNA or a single monoclonal antibody targeting with pshRNA in the treatment of GLC-82 lung cancer cells was more effective than without the ligand (Hou et al., 2016). Other albumin nanospheres were co-loaded in SPION as a vector of anticancer genes, modified by adding the anti-EGFR ligand monoclonal antibody with cetuximab as a targeting agent for adsorption of pDONR233-IFNG, aiming at the lung cancer cell line GLC-82. In this study, photo transfection and agarose gel electrophoresis were employed to demonstrate albumin nanosphere encapsulation. The Cell Counting Kit-8 test demonstrated that the combination therapy group had a more significant therapeutic impact on GLC-82 cells than the other treatment groups, notably a result of the increase in apoptosis and the ability to distribute and target efficiently (Zhang et al., 2015).

To improve the efficacy of doxorubicin in lung cancer, SPION was synthesized containing the chemotherapeutic agent doxorubicin and the anti-EGFR ligand cetuximab. Doxorubicin and cetuximab were conjugated using Fe_3_O_4_ magnetic nanoparticles to a previously conjugated dextran. *In vitro* studies on the A549 cells revealed that these nanoparticles dramatically suppressed cell growth more efficiently compared to NPs without anti-EGFR (Zhang et al., 2019). Other anti-EGFRs were used to target SPION in lung cancer. The Fe_3_O_4_ was loaded in PLGA-PEG-aldehyde nanoparticles synthesized by double emulsion method (water-in-oil-in water) and anti-EGFR conjugated on the surface by aldehyde-amine reaction. It was reported that these solid nanoparticles detect lung cancer efficently through magnetic resonance (Salehnia et al., 2019). Other SPION formulations were conjugated with oleic acid and carboxymethyl dextran and CD44v6 monoclonal antibody with Fe/carboxymethyl dextran ratio of 1/1 and 2/1 (w/w). The results indicated that these nanoparticles were strongly associated with A549 cell line *in vitro* and improved detection on magnetic resonance imaging (MRI) (Wan et al., 2016).

### 5.4. Lipid based nanoparticle

Lipid-based nanoparticles are nanoparticles composed of lipids which are usually in the form of liposomes, (Ninomiya et al., 2014) solid lipid nanoparticles, (Pindiprolu et al., 2019) or nanostructured lipid carriers (Haron et al., 2018). These nanoparticles are getting a lot of interest in drug development and cancer treatment. Some of these nanoparticles can transport both hydrophobic and hydrophilic compounds with extremely low or no toxicity. Furthermore, by possessing a prolonged half-life and regulated drug release, these nanoparticles can enhance the therapeutic action time. Lipid nanoparticles have been widely utilized as therapeutic carriers, particularly in cancer treatment. These nanoparticles have also been produced for active targeted distribution by conjugating them with ligand monoclonal antibodies that are selective for specific receptors.

To overcome hypoxia induced medication resistance in lung cancer, Li et al. (2017) developed complex liposomes that can transport oxygen and molecular targeted drugs. To co-administer erlotinib and PFOB (Perfluorooctyl bromide) against the EGFR overexpressed in NSCLC, anti-EGFR aptamer-conjugated chitosan-immobilized liposomes were constructed. Controlled drug release and anti-EGFR ligands were found to potently inhibits cell proliferation, induces apoptosis and improve cellular absorption when compared to nanoparticles without ligands (Li et al., 2017).

The synthesis of lipid-based nanoparticles was modified by utilizing an anti-EGFR aptamer to distribute erlotinib and surviving-shRNA. Chloroquine (CQ) was used with AP/ES (anti-EGFR Aptamers- modified polyamidoamine) to restore tumor vasculature to provide optimal drug/gene delivery and overcome treatment resistance in NSCLC cells. These nanoparticles showed good biostability, a controlled release profile, and a remarkable selectivity to EGFR, which is widely expressed in NSCLC. The improved drug delivery may increase the efficiency of the nanoparticles against lung cancer cell types. Both *in vitro* and *in vivo*, the combination with chloroquine displayed promising benefits against erlotinib-resistant cancer cells. In this study, the results showed that the IC50 of the nanoparticles in PC9 cells was much lower compared to H1975 cells, consistent with previous reports that erlotonib may affects this molecular targets and inhibits EGFR-mutant PC9 cells but fails to achieve good results NSCLC cells with EGFR T790M mutation are obtained (Lv et al., 2018).

Lipid nanoparticles encapsulating PD-L1 ligand antibodies were created using double emulsion and thin-film dispersion techniques to entrap Adriamycin. A549 cells were used for *in vitro* and *in vivo* studies. The results showed that at the same Adriamycin concentration, the intracellular derived fluorescence of the nanoparticles with this ligand was much larger than that of the nanoparticles without the ligand. The findings showed that nanoparticles containing an anti-PD-L1 ligand decreased tumor volume in the experimental animals more than the Adriamycin-only group (Xing et al., 2020). Other formulations of lipid-based nanoparticles using hyaluronic acid (HA) nanoparticles hybrid pH sensitive lipid-polymer with erlotinib and bevacizumab ligands for lung cancer targeting NSCLC exhibits stable nanoparticle. This formulation did not show dramatic changes in appearance, visible aggregation or precipitation, and no major changes were seen in particle size, zeta potential, and entrapment efficiency. Compared to nanoparticles without ligands, these nanoparticles showed an increase of accumulated particles in tumor tissue and low toxicity. The tumor volume in experimental animals was smaller in the group treated with ligand-containing nanoparticles (229.2 ± 13.1 mm^3^) than with ligand-free nanoparticles (437.3 ± 25.3 mm^3^). Therefore, this system is promising for NSCLC therapy (Pang et al., 2020).

### 5.5. Mesoporous silica and silica nanoparticle

Mesoporous silica is a type of nanoparticle with chemically and thermally stable nanomaterials with controlled morphology and porosity (Trewyn et al., 2007). This nanoparticle has been widely utilized and developed in drug delivery for lung cancer therapy. The addition of ligands to these nanoparticles can improve the system’s selectivity. The synthesis of silica nanoparticles was achieved by conjugating them with monoclonal antibodies. It was created by the self-assembly of mesoporous silica on reduced graphene oxide nano sheets with nanogap-aligned gold nanoparticles (AuNPs). It was then encapsulated and distributed within the nano pores of the mesoporous silica layer. Rhodamine 6 G (R6G) was then encapsulated into nano networks with anti-EGFR conjugated on the surface of the nanocomposite, along with PEG (polyethylene glycol). CPSS was a porous carbon silica nanofilm. When compared to the conventional cell line, the results revealed that these nanoparticles targeted overexpressed EGFR lung cancer cells (A549) with great specificity compared to (MRC-5) cells. The synergistic effect of linked AuNPs and nanosheets enables good photothermal therapeutic efficiency with a low power density (0.5 W cm^2^) of near-infrared laser. These findings imply that these nanoparticles constitute a one-of-a-kind theragnostic nano system with exceptional cell targeting and cell tracking capabilities, as well as photothermal therapeutic potential (Chen et al., 2016).

To detect lung cancer, silica nanoparticles containing anti-EGFR were coupled with NIRF (near-infrared fluorescent dye) and MB (methylene blue). This Anti-EGFR enhanced the cellular absorption *in vitro* and *in vivo* in experimental mice with tumor volume about 80–100 mm^3^ developed using A549 cells. It reveals that when anti-EGFR are used, these nanoparticles have excellent targeting capabilities (Wan et al., 2017). The manufacture of mesoporous silica in combination with anti-EGFR in the form of cetuximab was also carried out to deliver siRNA against polo-like kinase 1 (PLK1), which is critical in lung cancer mitosis. This EGFR response has been shown to be exhibited in up to 50% of lung cancer patients. *In vivo* experiments on A549 cancer-inolculated rats with tumor size of 120 mm^3^ indicated a reduction in tumor formation as well as the ability to function as an NSCLC targeted agent (Reda et al., 2019).

### 5.6. Others

The synthesis of functionally assembled supramolecular nanoparticles was performed for RNAi agent loading and tumor target treatment. The adamantane-grafted poly(ethylene glycol) molecule was modified with the specific binding ligand EGFR GE11 or the pH-sensitive fusogenic peptide GALA, which was then used for self-assembly with the cyclodextrin-grafted branched polyethyleneimine (CD-PEI), adamantane-grafted polyamidoamine dendrimer (Ad-PAMAM), and DNA. These nanoparticles showed that beneficial peptides can enhance targeted cell binding, internalization, and endosome release. Furthermore, it leads to enhanced reporter gene expression and effective target gene silencing. Systemic administration can effectively lower intratumoral VEGF protein levels, limit angiogenesis, and considerably suppress tumor development in A549 xenografts (Lu et al., 2020).

The CD146 antibody ligand in combination with 1,4,7-triazacyclononane-triacetic acid has been studied. PET imaging was employed as an experimental animal to examine tumor biodistribution and uptake of these nanoparticles in lung cancer-induced nude mice (A549, NCI-H358, NCI-H522, HCC4006, H23, and NCI-H460 cell lines). The relationship between CD146 expression and nanoparticle uptake was studied using graphical tools. *Ex vivo* biodistribution and immunohistochemical assays were performed to corroborate the correctness of the PET results and the spatial expression of CD146. When compared to other lung cancer cell lines, the H460 and H23 cell lines showed the highest expression of CD146, resulting in much greater cellular uptake in these cell lines (Sun et al., 2016).

Another nanoparticle development for lung cancer therapy was carried out by Abd-Rabou and Ahmed (2019), who successfully formulated doxorubicin, bevacizumab and CCR2 inhibitor in the form of nanoparticles. The results of this study showed a decrease in the viability of A549 cells and was able to increase the level of apoptosis in cell lines (Abd-Rabou & Ahmed, 2019). The formulation using an active substance in the form of gemcitabine which was formulated into nanoparticles with anti-EGFR ligands (SDP-GEM/PEI-PEG-anti-EGFR) indicated an increase in the eradication of cancer cells *in vitro*. This system can deliver drugs to the appropriate target for 3 hours *in vivo*, besides that it is able to extend lifetime and reduce tumor volume (Tang et al., 2020).

## 6. Author perspective

**Figure 4.**
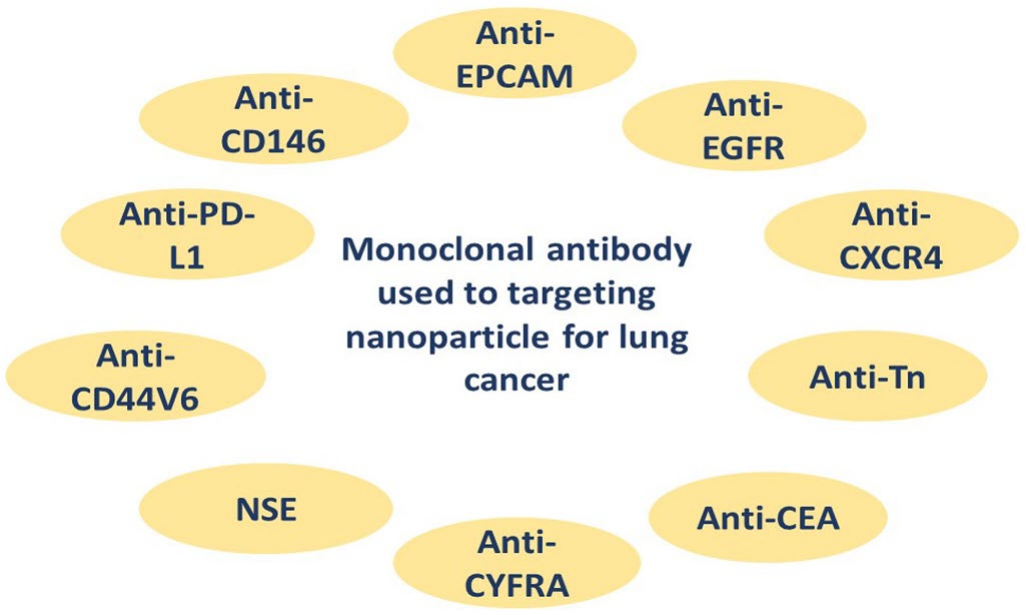
Types of monoclonal antibody used as targeted nanoparticles for lung cancer.

A monoclonal antibody is a kind of ligand utilized in active targeted drug delivery systems, particularly at cancer areas. Many cancer cells express antigens on their surfaces, which can be utilized as markers to deliver medications to the target area via specific antibodies. Nanoparticles containing this ligand have been created to treat cancer, especially lung cancer. Monoclonal antibody that has been used for targeting lung cancer are summarized in Figure 4. Monoclonal antibodies are used as ligands to help target nanoparticles to a specific population of cells, through exploiting the presence of specific antigens that are widely distributed on the surface of lung cancer cells (Tiseo et al., 2008). The binding reaction between the ligand and its antigen triggers endocytosis within the cell, resulting in rapid entry of the nanoparticles into cancer cells, as shown in Figure 3.

**Figure 5.**
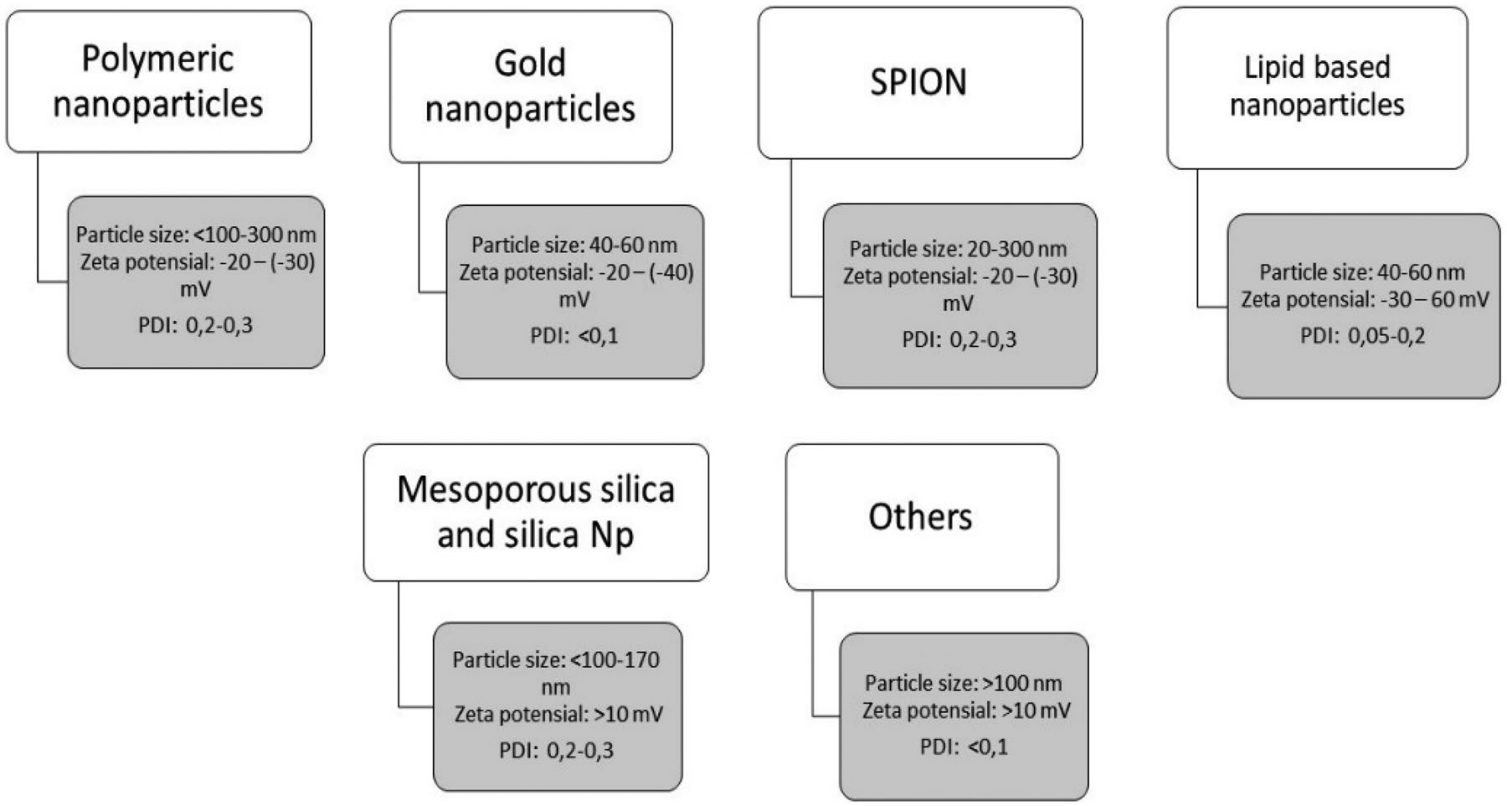
Characteristic of each type of nanoparticles.

Several types of nanoparticles have been used, including lipid-based, polymeric, SPION, dendrimers, mesoporous silica, and others (Figure 5). When conjugated to monoclonal antibody, anticancer drugs are most often attached to carboxyl or amino groups. The steps involved in conjugating chemotherapeutic drugs to monoclonal antibody often involve neutral binding, which can reduce antibody solubility and lead to aggregation and precipitation. Attention should also be paid to the number of drug residues attached to each monoclonal antibody. Ideally, the ratio of residual drug per monoclonal antibody should be maximized while maintaining acceptable levels of monoclonal antibody activity and specificity (Pietersz et al., 1987).

Many of the applications of these nanoparticles with monoclonal antibodies are still limited to cancer detection tests, while others have not yet been used to deliver specific active chemicals. Moreover, production of nanoparticles with antibodies to target the specific antigen is still of little use for positively targeting nanoparticles to lung cancer, such as Anti-EpCAM, anti-NSE, anti-ganglioside, etc. As a result, continued development of nanoparticles containing monoclonal antibodies is predicted to be successful as a medication delivery mechanism for lung cancer.

## 7. Conclusion

Lung cancer-specific ligand monoclonal antibodies are often used to produce polymeric nanoparticles, SPION nanoparticles, lipid-based nanoparticles, and others. Using these nanoparticles, drug is released in a controlled manner. Nanoparticles can also increase therapeutic effectiveness by enhancing drug accumulation at the target site. Recently, nanoparticles have been widely used for cancer. They are being used for more than just drug delivery; they are also being used to diagnose cancer and track its progression throughout the body. Targeted delivery systems in lung cancer therapy have been found to deliver drugs selectively, increase selectivity, and reduce the side effects of treatment. Therefore, the novel formulation of nanoparticles containing monoclonal antibodies as a targeted drug delivery system for lung cancer is interesting, and are continually developed.
